# Diagnosis through prisms: Unraveling its complexity

**DOI:** 10.1111/acem.15120

**Published:** 2025-02-05

**Authors:** Pat Croskerry, Mike Clancy

**Affiliations:** ^1^ Department of Emergency Medicine, Faculty of Medicine Dalhousie University Halifax Nova Scotia Canada; ^2^ Emergency Department, NHS Foundation Trust University Hospital Southampton Southampton UK

## Abstract

Following a review of accepted submissions for this special issue of the Society for Academic Emergency Medicine (SAEM)'s collected papers on diagnosis, we offer a commentary on the variety of reports. We use the metaphor of Newton's demonstration that a complex percept like the rainbow can be broken down by prisms, into a collection of different wavelengths of light. Like Feynman, we believe that the beauty of something may be revealed and augmented by reducing it to its constituent parts.

All research involves asking specific questions filtered through various prisms. Each may reveal some part of the answer to questions about complex phenomena, such as the diagnostic process. Slowly but gradually, by repeatedly asking our questions, we may improve our understanding of the sum of these parts.

In *Unweaving the Rainbow*, the evolutionary biologist Richard Dawkins addresses Keat's criticism (albeit light‐hearted) of Isaac Newton, who used prisms to demonstrate that white light was made up of all colors of the rainbow.^1^ By this simple act, the separation of light into different wavelengths, Newton had deprived the rainbow of its beauty and poetry. Similarly, though perhaps less light‐hearted, an artist friend of the Nobel laureate physicist Richard Feynman, commenting on the beauty of a flower, maintained that, as he was an artist, he could appreciate its beauty, whereas scientists “take it all apart making it a dull thing.” Feynman responded with: “First of all, the beauty that the artist sees is available to other people and to me too. Although I may not be quite as refined esthetically as he is, I can appreciate the beauty of a flower. At the same time, I see much more about the flower than he sees. I could imagine the cells in there, the complicated actions inside, which also have a beauty.”^2^ Like Dawkins, he acknowledged that reductionism could unveil a latent beauty, discovering poetry in the patterns of nature. For many of us, the imperative to make a diagnosis is often challenging, arduous, and occasionally uncomfortable. Yet, unraveling its complexity can reveal its beauty, as we expose its many parts. But, whereas Newton's unweaving of the rainbow was a relatively straightforward process, unraveling the diagnostic rainbow is considerably more challenging. No single physical property can explain its complexity.

Each study submitted to this issue of the journal satisfies Feynman's insistence that we conduct experiments to find out how something works, and each will contribute to the overall understanding of the diagnostic process. Every little bit helps. Like quantum mechanics, we must develop insight into its many and varied characteristics and search for ground rules that can be passed on to others. We have come to appreciate that the diagnostic process is both complex and complicated, reflected here in the diversity and depth of these collected papers, and pointing out the difficulty of achieving consensus on where our research efforts should be directed.

Over 60 independent variables have been identified in the diagnostic process, each with linear and nonlinear properties, and capable of interacting with each other.^3^ Described as a complex sociotechnical system, our task is to pass it through numerous and varied prisms to reveal its complexity and expand our understanding.

Diagnosis is the central process of medical practice—its spine—and reflects the complex interplay of the individual clinician's knowledge and experience and how it relates to the patient in front of them and their context. Given this, it is inevitable that there is variability in the practice of making a diagnosis with consequent errors; understanding such variability offers opportunities for improvement.

Diagnostic expertise in emergency medicine (EM) depends very much on deliberate practice in that environment, and adaptive expertise (a more comprehensive form of expertise) is achieved with an across‐the‐board knowledge base coupled with a common‐sense approach towards making rational decisions. There are a dozen or so ways in which rationality may be defined,^4^ and emergency clinicians can take their pick which to follow. It is likely that we fall into the phronetic one that Montgomery describes.^5^ Phronesis is a type of wisdom or intelligence that is concerned with practical action. It is often translated as “practical wisdom” or “prudence.” In their approach toward the diagnostic process, prudent emergency clinicians gravitate toward a diagnosis (or nondiagnosis) that is the best that can be made, under the prevailing context, given the available resources, and that works for the patient. In some settings (typically those in which billing issues are not at play), they can indicate their uncertainty, for example, chest pain NYD (not yet diagnosed) and avoid the trap of premature closure.

There are a number of papers that highlight such variability and should cause us to pause and think. The paper “Low sexually transmitted infection (STI) screening and presumptive treatment and high STI positivity among United States females visiting the emergency department after sexual assault” shows that our diagnostic decisions (in this case to test and presumptively treat) vary with significant consequences for the patients. The paper “Exploring diagnostic stewardship in the emergency department (ED) evaluation of pediatric abdominal pain in a statewide quality collaborative” highlights wide variation in cross sectional imaging rates, the importance of the quality of ultrasound assessment, and how imaging can be delayed if the patient is being transferred to a pediatric ED.

Another important feature of the diagnostic process, not always appreciated, is the manifestness of the disease itself. Few diseases present pathognomically with a discrete signal and no accompanying noise, “noise” meaning not only the acoustic variety but also noise in any other sensory modality, including the casual comment from a medical or nursing colleague. There is a continuum of manifestness that runs from high to low signal:noise ratio and, not surprisingly, we have less difficulty recognizing high signal:noise conditions.^3^ The challenge of diagnosing conditions that are not always manifest is evident in the report of “Factors and outcomes associated with under and overdiagnosis of sepsis in the first hour of emergency care,” which starts to unpick associations with missed diagnosis.

Finding cases we can learn from is not always easy and using triggers to identify potential cases that then leads to review of the case, can usefully identify where errors have occurred and learning opportunities exist. This is described in “Epidemiology of diagnostic errors in pediatric emergency departments using electronic triggers.” We should remind ourselves that errors, as psychologists see them, are aspects of behavior from which we can learn, and should not be seen pejoratively. The study is a welcome development as understanding the diagnostic process has always been hampered by the absence of a reliable trigger tool for diagnostic failure. Without such feedback, calibration of decision making is impaired.

Poor feedback is not uncommon in individual practice, and we miss the opportunity to rethink how we gather information, evaluate it, and add to it by undertaking more tests. The opportunity to recalibrate our thinking is often missed. Effective feedback needs to be reliable, accurate, and prompt. It is helpful if those providing it acknowledge the benefit they have of hindsight and that outcome bias does not interfere—the *error made in evaluating the quality of a decision when the outcome of that decision is already known*. When providing feedback, we should keep in mind that a lack of knowledge about outcome is often interpreted as a favorable outcome.^6^

The interface between the patient and the clinician is central to the diagnostic process and is often the scene of precursors to diagnostic failure. Barriers to that communication and how they can be overcome are important in error reduction, and these papers highlight important aspects of that communication: “Characterizing Spanish‐speaking patients centered care experience in the emergency department” (Acad Emerg Med. 2025 Jan;32(1):32‐44) and “Communication barriers to achieving optimal access to emergency rooms according to deaf and hard‐of‐hearing patients and health care workers: A mixed‐methods study.”

Seeing the patient as a partner rather than simply as an information provider to unravel the diagnostic puzzle, and accept their diagnosis and treatment plan, would enable their views to be more readily incorporated. This is especially important when there is genuine uncertainty about the diagnosis, and tests that can help can also be harmful. The article “Systematic review of barriers, facilitators, and tools to promote shared decision making in the emergency department” shows that patient decision aids increased shared decision making, tended to increase patient knowledge, and decreased decisional conflict and healthcare service usage.

Coming to terms with diagnostic inaccuracy is conditional on having a clear understanding of what we are talking about. This requires agreement on diagnostic error in general and how it should be defined. This should reflect the reality of EM practice, and this is addressed in the thoughtful piece “The problem with how we view medical (and diagnostic) error in emergency medicine.” Describing the ED as “dysfunctional, deficient, poorly designed, and highly stretched” with “limited resources and uncertain information,” the authors identify it as a unique, complex sociotechnical system that is defined by its people and everything else that is not people. Two particularly hazardous areas are identified: time pressures to make rapid diagnoses and transitions of care. Noting that expertise on decision making in the ED is poorly understood and underestimated will require a look outside health care for expertise. They quote John Senders, a professor of industrial engineering and psychology and expert on safety and human error: “Errors in medicine and the adverse events that follow are problems of psychology and engineering, not medicine,” a sentiment that appears to resonate well with the findings of the recent AHRQ report on diagnostic errors in the emergency department,^7^ where root causes of ED diagnostic errors were found to be mostly cognitive errors linked to the process of bedside diagnosis. While the availability of cognitive psychologists for ED work may be limited at present, the findings from their research on decision making can be readily applied to the decision making that occurs in that arena. At the same time, we also need agreement about the nitty gritty detail of individual condition definitions such as what constitutes a diagnostic failure in the context of PE, covered in “Ruling out pulmonary embolism safely: standardized reporting of the failure rate.”

As well as better definitions, we need practical help to deal with decision making in overcrowded departments, coping with the range of conditions we care for, and effectively using the often limited and fragmented information that may be available. How AI can help us out in general is well covered by “Leveraging artificial intelligence to reduce diagnostic errors in emergency medicine,” where it is described in three roles (information gathering, clinical decision support, and feedback through quality improvement). This offers insight into how AI can augment rather than threaten human cognition, providing that its outputs are always thoughtfully scrutinized, arguably in the same way as we should monitor the output of our own System 1 processes. How AI can help with some very specific problems is also addressed in “Using natural language processing to identify emergency department patients with incidental lung nodules requiring follow‐up.”

Inevitably when diagnostic error is identified, the conclusion is often that our thinking is at fault, so we have to come to terms with the current limitations of our insight into the workings of that black box that is our brain. We are sense‐making creatures, and the need for a workable theory becomes necessary if we are to understand what happens, why it happens, and how it can be improved. Answers to these questions have traditionally come from fields outside of medicine (psychology, neurosciences, and behavior), and so it has been slow to arrive in the medical orbit.

The dual process theory (DPT) has been widely accepted as such a workable theory, and there is now a broad consensus on two types of processes. That one system may supervise the other, or acts as a filter of the other, is the dominant view but the difficulty with delivering hard evidence about a process that is mostly invisible (cognition) inevitably means that alternative views emerge. “Diagnostic reasoning and cognitive error in emergency medicine: implications for teaching and learning” suggests that it is less the cognitive processes that are directly at fault in diagnostic errors but limitations in the knowledge that powers them and suggest how this can be mitigated. This runs counter to the observations from several studies, that knowledge deficits are rarely the final vector for diagnostic failure but much depends on how knowledge is defined. We concur that knowledge is not simply of the anatomy–physiology–pathology type, the main focus of medical training, but includes that acquired through deliberate practice in the setting of clinical medicine. Given that rationality is the defining feature of quality decision making, and that cognitive biases are considered by psychologists to be the greatest threat to our rationality, we must include knowledge of them and how to mitigate them. While this is often acquired through brutal experience, better perhaps to provide explicit training, so they are less mysterious when they do come along.

Further, as noted in this paper, we should not polarize Systems 1 and 2 unduly but see them instead as essential, mutual, complementary components of our decision making, each providing adaptive value.^8^ Recent studies in comparative psychology have shown that nonhuman species, including insects, exhibit DPT processes in their decision making,^9^ so evolutionary pressures may have led to a selection of dual processing, with Type 1 processes evolving first and Type 2 later. Ontogeny can be seen to recapitulate phylogeny in that this is how it seems to work in practice; our attention is first grabbed by System 1, but if any refinement of response is required, it may be provided by System 2. The trick comes in knowing when that intervention is required. Overall, the advantage that humans have with advanced language allows for a sophisticated interaction between the two systems.

What is important is that thinking about thinking becomes mainstream, and what works in the real world is identified along with its inherent uncertainties. Figuratively, the cognitive interface between clinician and patient, virtual or otherwise, is the motherlode where investigators can mine significant gains in understanding the diagnostic process. An example of such a pragmatic approach is reported in “Virtual patient and feedback intervention to improve clinical reasoning for dizziness in the emergency department,” which demonstrated improved diagnostic accuracy and appropriate CT utilization and retention of skills 6 months later.

While we may argue about the accuracy and definition of diagnostic error, there is no doubt that it is a reality and that current error rates of 10% or more in EM suggest that the problem is real and not going way. No airline or engineering company could continue with such rates. In a medical world with increasing factual knowledge, we need to look to new ways of handling that knowledge (that does not involve holding it in our heads) yet at the same time understand better how to handle that knowledge effectively, recognizing when it can go wrong and preempting adverse outcomes. Given the difficulty in reducing this error, it is understandable that there are calls to view the problem differently and two papers address this: “The problem with how we see medical and diagnostic error in emergency medicine” and “From diagnostic errors to diagnostic excellence in emergency care: time to flip the script.”

It seems that we have come a long way from the epic, *fin de siècle* Institute of Medicine report *To Err is Human* in 1999,^10^ which represented both a tipping point and a turning point. Besides ushering in the modern era of patient safety, it steered the focus away from reflexive blaming habits when things went wrong, instead putting the emphasis on the system. We now appear to be at a new tipping point that deflects back to the individual, at least so far as their cognition is concerned. We recognize that understanding cognition provides insight into most of our mistakes—with the exception of spinal reflexes, all behaviors are preceded by cognition, ranging from a reflexive, subconscious act (intuition) all the way through to the purposeful deliberations of System 2 processes. It remains true that the system may contribute to many significant error‐producing conditions: inverted‐U functions are common showing how human brain performance responds to various constraints such as stress, workload, cognitive load, fatigue, sleep deprivation, dysphoria, and other factors, but we can now describe these deficits more quantitatively in terms of impairment of frontal lobe function and the associated loss of cognitive control and executive function. Recent studies have investigated this at a molecular level implicating the neurotoxic effects of extracellular fluid glutamate in impaired cognitive function.^11^

That there is a wide interest and energy in the diagnostic process is evidenced by the range of papers accepted for publication in this issue of *AEM* as well as those that were rejected. The search for prisms and filters that refine our knowledge of the diagnostic process goes on, and any study that reduces uncertainty will be useful.

## CONFLICT OF INTEREST STATEMENT

The authors declare no conflicts of interest.

## Data Availability

Data sharing not applicable to this article as no datasets were generated or analysed during the current study.

## References

[1] Dawkins R . Unweaving the Rainbow. Houghton‐Mifflin; 1998.

[2] Feynman RP . The Pleasure of Finding Things Out: The Best Short Works of Richard P. Feynman Paperback. Basic Books; 2005.

[3] Croskerry P , Campbell S , Petrie D . The challenge of cognitive science for medicine. Cogn Res Princ Implic. 2023;8:13. doi:10.1186/s41235-022-00460-z 36759370 PMC9911579

[4] Djulbegovic B , Elqayam S . Many faces of rationality: implications of the great rationality debate for clinical decision‐making. J Eval Clin Pract. 2017;23:915‐922. doi:10.1111/jep.12788 28730671 PMC5655784

[5] Montgomery K . How Doctors Think. Clinical Judgment and the Practice of Medicine. Oxford University Press; 2006:165‐166.

[6] Croskerry P . The feedback sanction. Acad Emerg Med. 2000;7:1232‐1238. 11073471 10.1111/j.1553-2712.2000.tb00468.x

[7] Newman‐Toker DE , Peterson SM , Badihian S , et al. Diagnostic Errors in the Emergency Department: A Systematic Review. Comparative Effectiveness Review No. 258. (Prepared by the Johns Hopkins University Evidence‐Based Practice Center under Contract No. 75Q80120D00003). AHRQ Publication No. 22(23)‐EHC043. Agency for Healthcare Research and Quality. 2022. 10.23970/AHRQEPCCER258 36574484

[8] Croskerry P , Petrie DA , Reilly JB , Tait G . Deciding about fast and slow decisions. Acad Med. 2014;89:197‐200. 24362398 10.1097/ACM.0000000000000121

[9] Kelly M , Barron AB . The best of both worlds: dual systems of reasoning in animals and AI. Cognition. 2022;225:105118. doi:10.1016/j.cognition.2022.105118 35453083

[10] Institute of Medicine (US) Committee on Quality of Health Care in America . To Err Is Human: Building a Safer Health System. National Academy Press; 2000.

[11] Friedman NP , Robbins TW . The role of prefrontal cortex in cognitive control and executive function. Neuropsychopharmacology. 2022;47:72‐89. doi:10.1038/s41386-021-01132-0 34408280 PMC8617292

